# Breaking the treatment dilemma of Schatzker IV fractures: finite element analysis validates hybrid single-plate with tension screw fixation for synergistic optimization of stability and minimally invasive outcomes

**DOI:** 10.3389/fbioe.2025.1650132

**Published:** 2025-10-31

**Authors:** Mingxiang Liu, Zulong Zhou, Chaofan Wu, Chaoqun Wu, Run Fang, Chengnan Zhang, Lingchao Kong, Rende Ning

**Affiliations:** Department of Orthopedics, The Third Affiliated Hospital of Anhui Medical University (The First People’s Hospital of Hefei), Anhui Medical University, Hefei, Anhui, China

**Keywords:** tibial plateau fracture, Schatzker type IV, finite element analysis, internal fixation strategy, minimally invasive, mechanical stability

## Abstract

**Objective:**

The core challenge in treating Schatzker type IV tibial plateau fractures lies in balancing mechanical stability with minimally invasive techniques. Traditional double-plate fixation carries a high risk of soft tissue complications, while single-plate fixation provides insufficient mechanical strength. This study aims to systematically evaluate and compare the biomechanical performance of five internal fixation strategies for Schatzker type IV-A and IV-B fractures using finite element analysis (FEA), exploring whether optimized fixation configurations can achieve synergy between minimally invasive treatment and stability.

**Method:**

Three-dimensional models of Schatzker type IV-A and IV-B fractures were constructed based on CT data from a 43-year-old male patient. Soft tissue models including ligaments and menisci were established. Five fixation methods were simulated: isolated medial plate (IMLP), medial plate with two posteromedial tension screws (IMLP + 2PMS), medial plate with two lateral tension screws (IMLP + 2LTS), posteromedial and medial double plating (PMP + MLP), and medial and lateral double plating (MLDP). Axial loads from 300 N to 2400 N were applied to simulate conditions ranging from standing to vigorous activity. Implant stress, tibial shaft stress, and fracture fragment micromotion were quantified.

**Result:**

Under a 1200 N load, hybrid fixation modes (IMLP + 2PMS and IMLP + 2LTS) demonstrated superior biomechanical performance. They exhibited the lowest peak implant stress (Type IV-A: IMLP + 2PMS 124.21 MPa; Type IV-B: IMLP + 2PMS 115.64 MPa), significantly better than the IMLP group (∼248 MPa), and comparable or superior to double-plate fixation groups (MLDP, PMP + MLP). While fracture fragment displacement showed no significant differences across all fixation methods, IMLP + 2PMS effectively reduced stress in type IV-B fragments. Regarding stress distribution in the tibial shaft, hybrid fixation provided a more uniform and physiological pattern compared to double-plate fixation. The results indicate differential responses to fixation strategies between type IV-A and IV-B fractures, with type IV-B deriving more pronounced benefits from posteromedial tension screws.

**Conclusion:**

The hybrid fixation configuration of a “medial plate combined with tension screws” represents a biomechanically optimal solution for treating Schatzker type IV-A and IV-B fractures. It provides stability comparable to double-plate fixation while significantly reducing implant stress concentration and the “stress-shielding” effect through a minimally invasive approach, achieving a synergy between minimal invasiveness and stability.

## 1 Introduction

Tibial plateau fractures, as common intra-articular injuries, account for approximately 1.2%–1.7% of all fractures and 5%–8% of lower limb fractures. Their disability rate and functional impact are significantly higher than those of other lower limb fracture types (Aubert et al., 2021; Reátiga Aguilar et al., 2022). Epidemiological studies indicate that such fractures frequently occur in young and middle-aged adults, mostly resulting from high-energy trauma (e.g., traffic accidents, falls from height), and are often accompanied by severe soft tissue complications (Reátiga Aguilar et al., 2022; Oladeji et al., 2020). This injury mechanism poses challenges for clinical treatment strategies—not only must mechanical stability be ensured, but minimally invasive techniques are also required to reduce postoperative incision complications, particularly in cases with poor soft tissue conditions.

The classic classification of tibial plateau fractures was proposed by Joseph Schatzker, who categorized these fractures into six morphological types based on fracture patterns and mechanisms (Kfuri and Schatzker, 2018). Among these, Schatzker type IV is a distinct high-energy injury pattern, accounting for approximately 10%–30% of all tibial plateau fractures (Chapman et al., 2023; Yang et al., 2012). It is characterized by a split or depression of the medial plateau, often caused by varus and internal rotation forces applied to the knee in a flexed position. This results in the femoral condyle impacting the medial tibial plateau, generating a coronal plane fracture line perpendicular to the long axis of the tibia (Markhardt et al., 2009). Unlike other Schatzker types, type IV fractures involve the weight-bearing zone of the medial column (which bears 60% of knee load) and the stability structures of the posterior column, often accompanied by coronal plane knee dislocation, significantly increasing the complexity of treatment (Ramoutar et al., 2019; Chen et al., 2016). Moreover, the most severe challenge in Schatzker type IV fractures lies in their extremely high rate of associated soft tissue injuries (Yan et al., 2021). Imaging and intraoperative exploration confirm ligament injury rates of 77%–100%, meniscal tear rates exceeding 80%, and a high risk of neurovascular damage (Risitano et al., 2025; Bormann et al., 2023; Peng et al., 2024). In addition to the above classification, the three-column tibial plateau classification proposed by Professor Congfeng Luo, based on CT fracture morphology, also highlights the unique pattern of these fractures (Luo et al., 2010; Zhu et al., 2012; Zhang et al., 2019; He et al., 2018), Specifically, Schatzker type IV fractures correspond to the “medial column + posterior column” injury pattern in the three-column classification. The medial column is critical for bearing 60%–70% of the knee’s static load, while the posterior column resists axial compression forces and maintains posterior knee stability (Zhu et al., 2012). A split of the medial column combined with compression of the posterior column can lead to varus laxity (evidenced by > 5 mm gap on stress radiographs). Therefore, investigating which internal fixation method can effectively stabilize both the medial and posterior columns is of significant clinical importance.

Conventional wisdom holds that plate-screw systems provide greater stability than screws alone, and combined plating offers excellent mechanical stability through multi-planar fixation. Consequently, double-plating (e.g., posteromedial and medial-lateral plates) is commonly used in clinical practice (Wei et al., 2023). From the perspective of the three-column fixation principle, this strategy aims to simultaneously stabilize both the medial and posterior columns, but at the cost of extensive surgical exposure. However, such extensive dissection increases intraoperative blood loss by approximately 40% and raises the risk of wound complications to 18%–25%, particularly in elderly patients or those with high-energy trauma and compromised soft tissue conditions (Stannard et al., 2010; Bennett and Browner, 1994). From the perspective of the three-column fixation principle, this strategy aims to simultaneously stabilize both the medial and posterior columns, but at the cost of extensive surgical exposure. However, such extensive dissection increases intraoperative blood loss by approximately 40% and raises the risk of wound complications to 18%–25%, particularly in elderly patients or those with high-energy trauma and compromised soft tissue conditions (Smith et al., 2024; Song et al., 2019).

Based on the location of the fracture line, Wahlquist et al. further classified type IV fractures into subtypes A, B, and C. Type IV-A refers to fractures where the fracture line originates medial to the intercondylar eminence, type IV-B refers to those originating within the intercondylar eminence, and type IV-C refers to fractures originating lateral to the intercondylar eminence (Wahlquist et al., 2007). It is noteworthy that type IV-C fractures extend into the metaphysis, compromising metaphyseal blood supply, and are often associated with intercondylar eminence avulsion, leading to an increased risk of combined cruciate ligament injuries and nonunion (Liu Z. et al., 2023). Therefore, type IV-C represents a high-risk subtype among type IV fractures and should be managed as a complex intra-articular fracture. Intraoperative management must address both bony structure and ligamentous stability, while preoperative CT and MRI assessments of the intercondylar eminence and ligament status are crucial for treating type IV-C fractures. Our research group has previously utilized finite element analysis (FEA) to compare in detail the biomechanical performance of five fixation methods for Schatzker type IV-C tibial plateau fractures. Additionally, we have conducted a series of studies focusing on X-ray and CT evaluation, as well as soft tissue injury-related issues in type IV-C tibial plateau fractures (Zhou et al., 2025; Liu Y. et al., 2023).

However, unlike type IV-C fractures, types IV-A and IV-B are characterized by fracture lines located medial to the intercondylar eminence, confined anatomically to the medial plateau, and share certain similarities in treatment. Biomechanical studies have shown that the lower limb mechanical axis passes precisely through the intercondylar eminence, and the medial tibial plateau bears higher mechanical loads compared to the lateral plateau. Consequently, the treatment of type IV-A and IV-B tibial plateau fractures demands higher requirements for mechanical stability. Furthermore, compared to type IV-C, the clinical incidence of types IV-A and IV-B is significantly higher (type A: 25%, type B: 42%, type C: 32%) (Wahlquist et al., 2007). Therefore, investigating the optimal internal fixation strategy for these subtypes holds substantial clinical importance.

Therefore, building upon this foundation, we once again applied the well-established finite element analysis (FEA) method to standardized models of Schatzker type IV-A and IV-B tibial plateau fractures. This study systematically evaluated and compared the biomechanical performance of the following five internal fixation strategies (Zhou et al., 2025): isolated medial locking plate (IMLP), medial locking plate with two posteromedial support screws (IMLP + 2PMS), medial locking plate with two lateral tension screws (IMLP + 2LTS), posteromedial plus medial locking plate (PMP + MLP), and medial-lateral dual plating (MLDP). By simulating five axial loading conditions (300 N, 600 N, 1200 N, 1800 N, and 2400 N) representing single-leg stance, double-leg stance, walking, running, and other vigorous activities, we quantitatively analyzed implant stress, tibial shaft stress distribution, and fracture fragment micromotion. The aim was to explore whether optimized fixation configurations could achieve mechanical stability comparable to double plating while significantly reducing surgical trauma and postoperative soft tissue complications, thereby striking a balance between mechanical stability, minimal invasiveness, and low complication rates.

The findings of this study will provide an objective and quantitative biomechanical basis for selecting internal fixation strategies in Schatzker type IV fractures. They will guide clinicians in identifying the optimal balance between achieving anatomical stability and minimizing surgical trauma and complication risks, ultimately improving patient outcomes.

## 2 Materials and methods

This study was approved by the Institutional Review Board of the Third Affiliated Hospital of Anhui Medical University (Hefei First People’s Hospital) (Approval Code: 2025-067-01). A 43-year-old male patient, weighing 75 kg and measuring 170 cm in height, who sustained a unilateral tibial plateau fracture due to a traffic accident and required lower limb CT examination, was selected from our hospital. After providing informed consent and signing a written consent form, the patient underwent CT scanning of the affected lower limb.

### 2.1 Construction of Schatzker type IV tibial plateau fracture models

**FIGURE 1:**
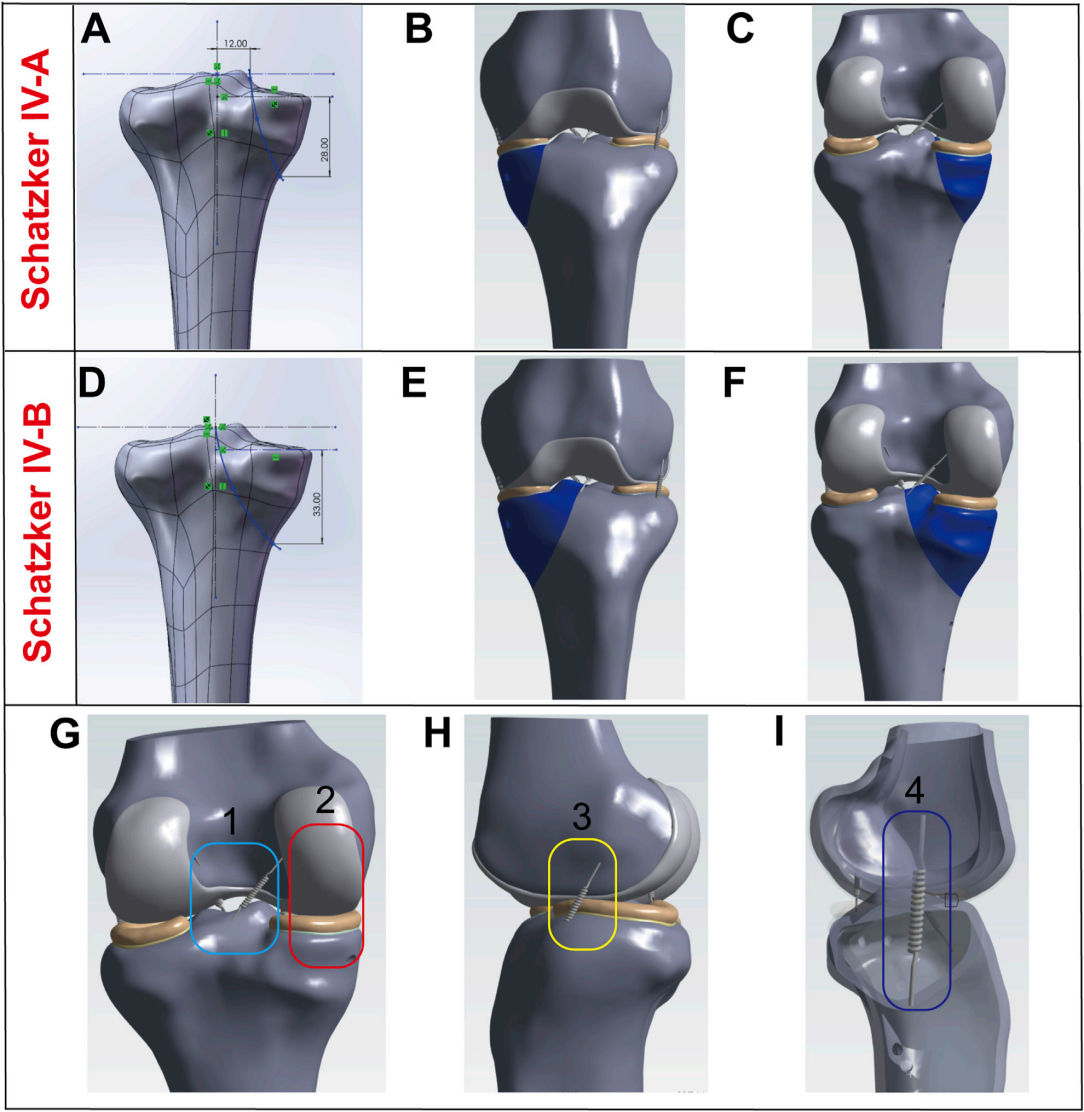
Model Establishment. **(A–C)** Establishment of the Schatzker type IV-A tibial plateau fracture model. **(D–F)** Establishment of the Schatzker type IV-B tibial plateau fracture model. **(G–I)** Modeling and visualization of soft tissues (ligaments and menisci): Ligaments are simulated using springs, where 1 represents the anterior and posterior cruciate ligaments, 2 denotes cartilage and the medial meniscus, 3 indicates the lateral collateral ligament, and 4 refers to the medial collateral ligament.

A Schatzker type IV tibial plateau fracture model was constructed using the patient’s CT image data. The specific steps were as follows: First, CT images including coronal, sagittal, and axial views were acquired and saved in DICOM format. Additionally, 3D model information of the distal femur and tibia was extracted from the CT data. These images were then imported into Mimics 21.0 software (Materialise, Belgium) for three-dimensional (3D) model reconstruction. Subsequently, the reconstructed 3D model was imported into Geomagic 2021 software (Geomagic, Inc.) for further smoothing and fracture line drawing. The Schatzker type IV-A fracture was characterized by a fracture line originating medial to the intercondylar eminence, with the proximal point of the fracture line located 12 mm horizontally medial to the intercondylar eminence and the distal point 28 mm vertically below the tibial plateau (Figures 1A-C). The Schatzker type IV-B fracture was defined by a fracture line originating within the intercondylar eminence, with the proximal point of the fracture line located at the intercondylar eminence (0 mm horizontal distance) and the distal point 33 mm vertically below the tibial plateau (Figures 1D-F).

### 2.2 Modeling of soft tissues (ligaments and menisci)

Ligaments were modeled as spring elements to enhance the realism of joint motion and reinforce the constraints between the femur and tibia. The insertion points of the ligaments were defined based on anatomical video data and existing literature (Lee et al., 2024). The medial collateral ligament (MCL) and lateral collateral ligament (LCL) were constructed as shown in Figures 1H,I. The anterior cruciate ligament (ACL) and posterior cruciate ligament (PCL) were modeled as illustrated in Figure 1G. The menisci were also incorporated into the model, with their geometry and positioning depicted in Figure 1G.

### 2.3 Grouping of fixation models for Schatzker type IV tibial plateau fractures

**FIGURE 2:**
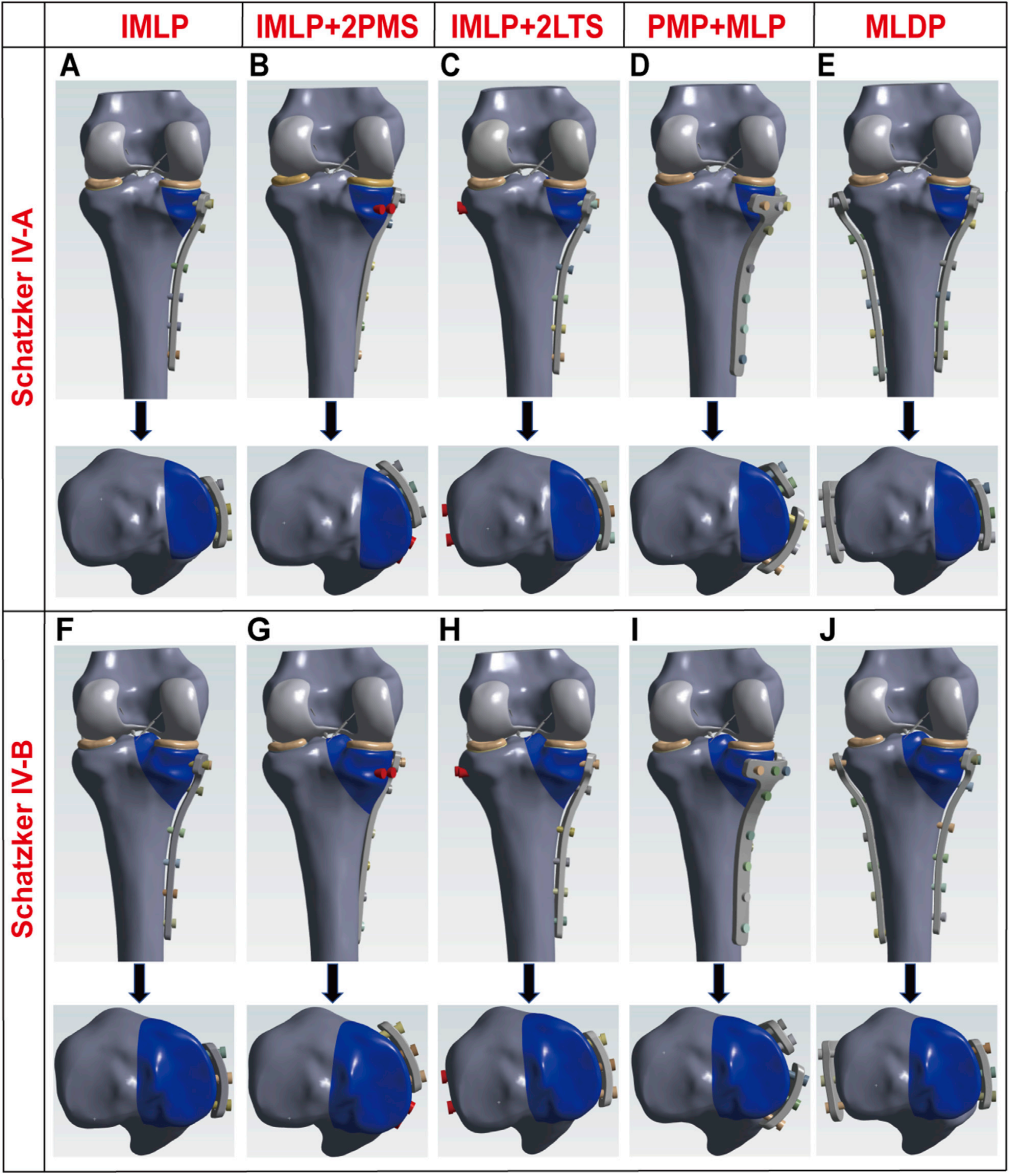
Three-dimensional schematic diagrams of five different internal fixation methods for Schatzker type IV tibial plateau fractures. **(A)** Isolated medial plate fixation for Schatzker type IV-A tibial plateau fracture. **(B)** Medial plate combined with two posteromedial tension screws for Schatzker type IV-A tibial plateau fracture. **(C)** Medial plate combined with two lateral tension screws for Schatzker type IV-A tibial plateau fracture. **(D)** Medial and posteromedial double plating for Schatzker type IV-A tibial plateau fracture. **(E)** Medial and lateral double plating for Schatzker type IV-A tibial plateau fracture. **(F)** Isolated medial plate fixation for Schatzker type IV-B tibial plateau fracture. **(G)** Medial plate combined with two posteromedial tension screws for Schatzker type IV-B tibial plateau fracture. **(H)** Medial plate combined with two lateral tension screws for Schatzker type IV-B tibial plateau fracture. **(I)** Medial and posteromedial double plating for Schatzker type IV-B tibial plateau fracture. **(J)** Medial and lateral double plating for Schatzker type IV-B tibial plateau fracture.

**FIGURE 3:**
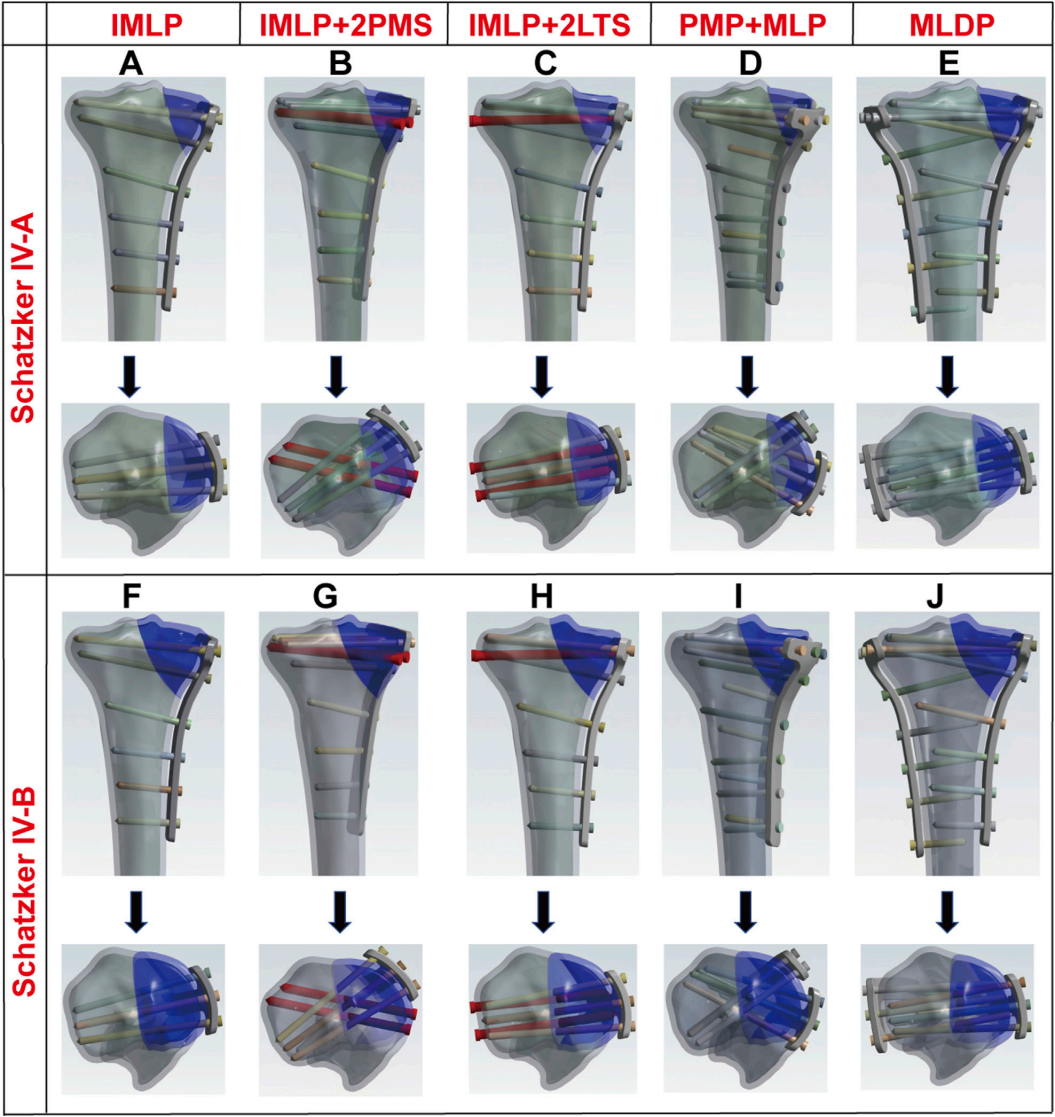
Radiographic schematic diagrams of plate and screw configurations for five internal fixation methods in Schatzker type IV tibial plateau fractures. **(A–E)** IMLP, IMLP + 2PMS, IMLP + 2LTS, PMP + MLP, and MLDP for Schatzker type IV-A tibial plateau fracture, respectively; **(F–J)** IMLP, IMLP + 2PMS, IMLP + 2LTS, PMP + MLP, and MLDP for Schatzker type IV-B tibial plateau fracture, respectively.

In this study, all internal fixation components were constructed based on technical parameters provided by the manufacturer (Shanghai Sanyou Medical Instrument Co., Ltd.) (Table 1). The experimental groups were established as previously described (Zhou et al., 2025). The specific experimental grouping schemes were as follows: IMLP Group: Comprised a medial T-shaped plate (placed on the medial side of the tibia) and 8 screws. The proximal 3 screws were inserted at a 5° angle to the tibial plateau, while the distal 5 screws were inserted at angles of 10°, 13°, 6°, 6°, and 1°, respectively. IMLP + 2PMS Group: Based on the IMLP structure, two 4.5 mm diameter tension screws were added to the anteromedial tibia (inserted at angles of 8°, 16°, 7°, 4°, 6°, and 1°). Additionally, two 4.5 mm tension screws were placed posteriorly, parallel to the tibial plateau, to enhance the stability of the posterior fracture fragment. IMLP + 2LTS Group: The medial plate placement followed the IMLP group, with the distal 5 screws inserted at angles of 11°, 14°, 7°, 7°, and 1°. Two 4.5 mm tension screws were placed laterally, parallel to the tibial plateau, to reinforce the fixation of the lateral fracture fragment. PMP + MLP Group: This group consisted of a medial T-shaped plate, a golf-shaped plate, and 15 screws. The golf-shaped plate was fixed to the anteromedial tibia, while the medial plate was fixed to the posteromedial tibia. The screw angles were set according to their specific insertion positions. MLDP Group: Utilized a medial T-shaped plate, a lateral L-shaped plate, and 16 screws. The medial plate was fixed to the medial tibia, and the lateral plate was fixed to the lateral tibia, with screw angles adjusted based on their fixation positions. Finally, the geometric models of Schatzker type IV-A and IV-B tibial fractures were integrated and assembled with various internal fixation models using SolidWorks 2021 software, forming the final research models as illustrated in Figures 2, 3 (Zhou et al., 2025).

**TABLE 1:** Parameters of internal fixation components (Zhou et al., 2025).

	Media T-plates	Golf steel plate	Lateral L-plate	Tension screw
Length	102 mm	102 mm	105 mm	—
Thickness/Diameter	3.5 mm	3.5 mm	3.5 mm	4.5 mm
Number of screws	8	7	8	—

**FIGURE 4:**
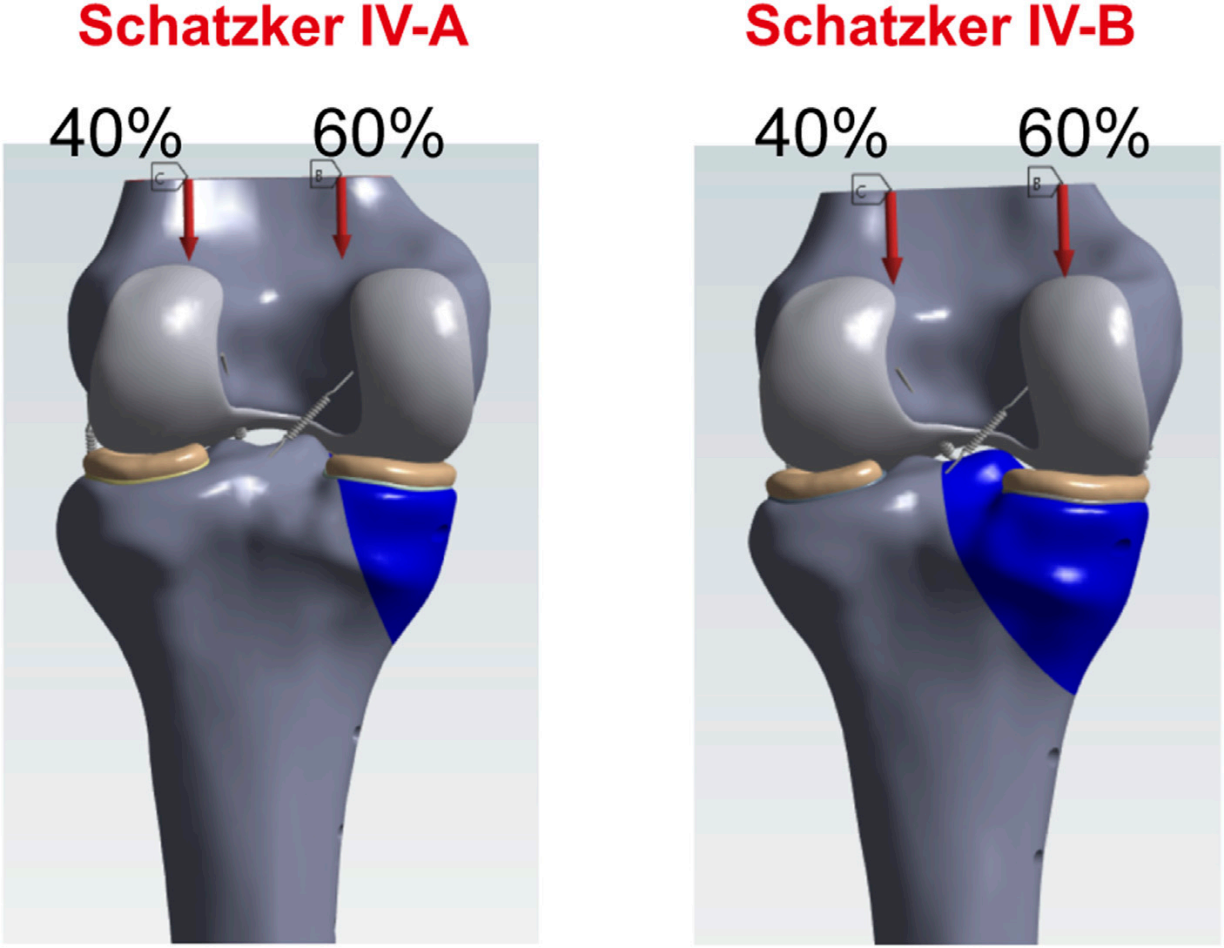
Loading and boundary conditions (Zhou et al., 2025).

It is noteworthy that although medial-lateral double plating (MLDP group) is less commonly used in the treatment of Schatzker type IV-A/B fractures, it was included in this study for two key reasons: (1) Historical Control Significance: Combined plating provides excellent mechanical stability through multi-planar fixation. Double plating (e.g., posteromedial and medial-lateral plates) has been widely adopted in clinical practice, and its biomechanical data can serve as a benchmark reference for evaluating hybrid fixation techniques. (2) Mechanical Comparison Requirement: Lateral plating offers the highest torsional stiffness, which helps quantitatively assess whether hybrid fixation (single plate + screws) can achieve comparable stability.

### 2.4 Material properties

The following material parameters were adopted for simulation analysis in this study: The titanium alloy implant model was assigned an elastic modulus of 110,000 MPa and a Poisson’s ratio of 0.3. Cortical bone was modeled with an elastic modulus of 17,000 MPa and a Poisson’s ratio of 0.3. Trabecular bone was assigned an elastic modulus of 5,000 MPa and a Poisson’s ratio of 0.3. Cartilage was modeled with an elastic modulus of 12 MPa and a Poisson’s ratio of 0.45. The menisci were assigned an elastic modulus of 80 MPa and a Poisson’s ratio of 0.3. For ligaments, a single spring stiffness was applied to each insertion site (Table 2). All materials, including implants and bone tissues, were assumed to be isotropic and linearly elastic (Zhou et al., 2025).

**TABLE 2:** Material properties (Zhou et al., 2025).

Components	Modulus of elasticity (MPa)	Poisson’s ratio
Cortical bone	17000	0.3
Cancellous bone	5000	0.3
Titanium alloy	110000	0.3
cartilage	12	0.45
meniscus	80	0.3

### 2.5 Loading and boundary conditions

During normal gait, the biomechanical load on the knee joint is approximately two to three times the body weight (Taylor et al., 2004), with the medial and lateral plateaus bearing about 60% and 40% of the load, respectively (Zhou et al., 2025). For a healthy adult weighing 75 kg, the compressive force on the tibial plateau during standing is calculated as follows:

75 kg × 9.8 N/kg×85.6% = 629.16 N (Gao et al., 2022). Walking and running exert forces on the tibial plateau that are two to three times greater than during standing (Tai et al., 2009). Accordingly, the following axial loads were applied to the models to simulate different activity levels: 300 N (bipedal standing), 600 N (single-leg standing), 1200 N (walking), 1800 N (running), 2400 N (vigorous activities). For all loading conditions, 60% of the load was distributed to the medial compartment to reflect physiological load distribution.

For modeling the plate-screw and screw fixation systems, the contact between implants and bone tissue was simulated using a Coulomb friction model with a friction coefficient of 0.3. The mechanical behavior of the locking screw mechanism was simulated using a node coupling method, which achieved rigid connections between plates and screws through shared common nodes. Additionally, to accurately characterize the mechanical interaction between the tibia and fibula, a fully bonded contact algorithm was employed to simulate load transfer mechanisms between them (Zhou et al., 2025).

### 2.6 Finite element analysis

This study conducted numerical simulations using the finite element analysis software ANSYS Workbench 2021 R1. Material properties were assigned to each component according to the parameters listed in Table 2. Following model importation, finite element meshing was performed for five experimental models with different configurations, with detailed node and element counts for each model provided in Table 3. After completing these preprocessing steps, loads were applied according to predefined boundary conditions. The static structural analysis module was employed to obtain key mechanical parameters, including stress distributions and displacement fields of the internal fixation system, fracture fragments, and tibial shaft. It should be specifically noted that all mechanical data in this study were derived directly from simulation results generated by ANSYS Workbench 2021 R1.

**TABLE 3:** Meshing of each mode (Zhou et al., 2025).

	Configuration	IMLP	IMLP + 2PMS	IMLP + 2LTS	PMP + MLP	MLDP
IV-A	Number of nodes	370808	404603	408625	475892	498856
	Number of elements	200801	219903	222192	260247	272844
IV-B	Number of nodes	370374	405896	408775	476358	499161
	Number of elements	200487	220705	222203	260453	272955

## 3 Results

**FIGURE 5:**
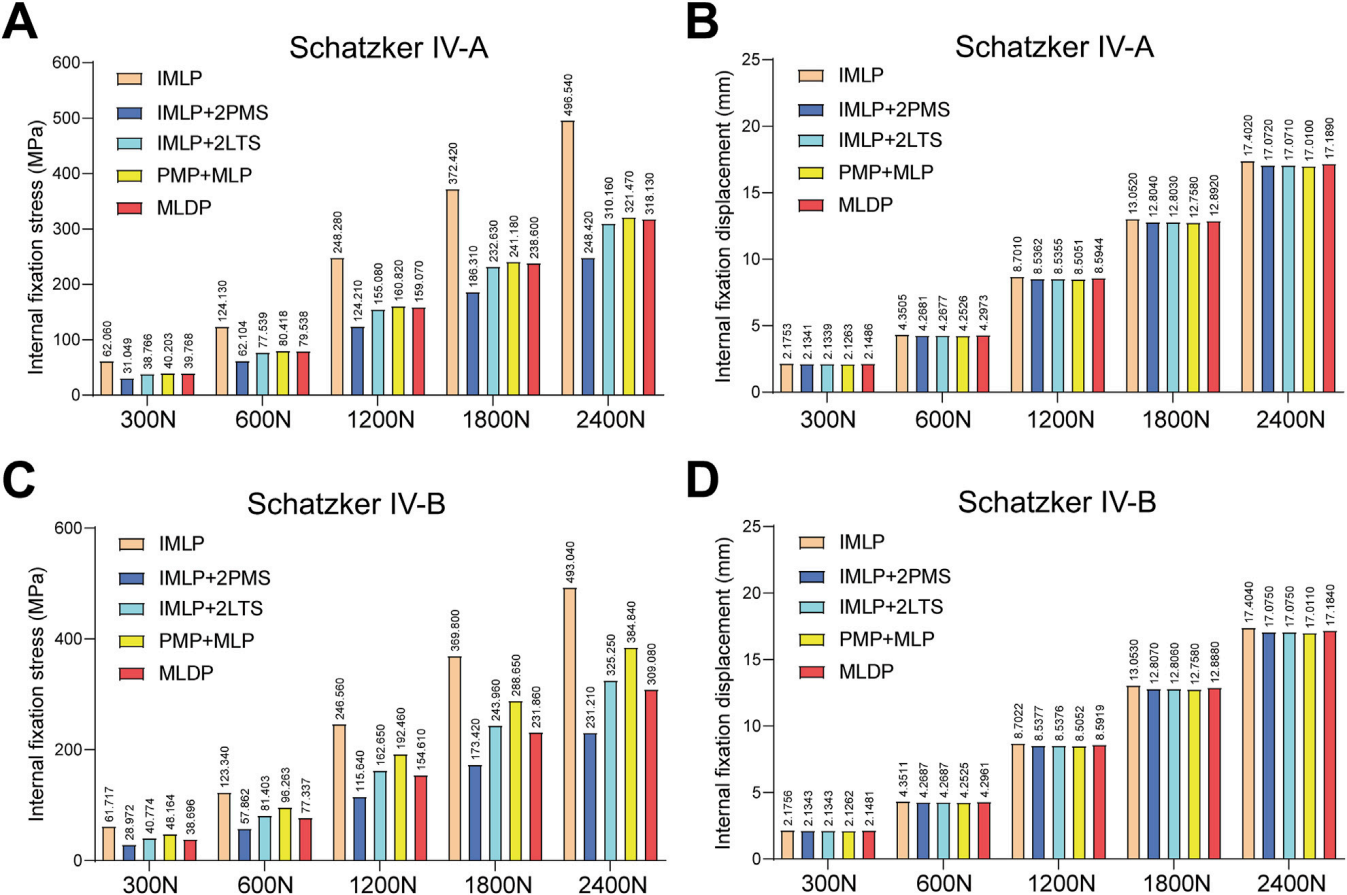
Statistical analysis of internal fixation stress and displacement under five axial loads (300 N, 600 N, 1200 N, 1800 N, and 2400 N). **(A)** Statistical graph of internal fixation stress for five fixation methods in Schatzker type IV-A tibial plateau fractures. **(B)** Statistical graph of internal fixation displacement for five fixation methods in Schatzker type IV-A tibial plateau fractures. **(C)** Statistical graph of internal fixation stress for five fixation methods in Schatzker type IV-B tibial plateau fractures. **(D)** Statistical graph of internal fixation displacement for five fixation methods in Schatzker type IV-B tibial plateau fractures.

**FIGURE 6:**
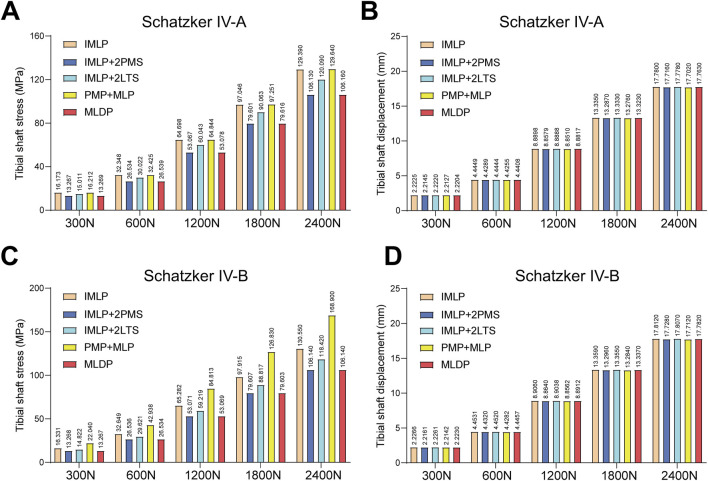
Statistical analysis of tibial shaft stress and displacement under five axial loads (300 N, 600 N, 1200 N, 1800 N, and 2400 N). **(A)** Statistical graph of tibial shaft stress for five fixation methods in Schatzker type IV-A tibial plateau fractures. **(B)** Statistical graph of tibial shaft displacement for five fixation methods in Schatzker type IV-A tibial plateau fractures. **(C)** Statistical graph of tibial shaft stress for five fixation methods in Schatzker type IV-B tibial plateau fractures. **(D)** Statistical graph of tibial shaft displacement for five fixation methods in Schatzker type IV-B tibial plateau fractures.

**FIGURE 7:**
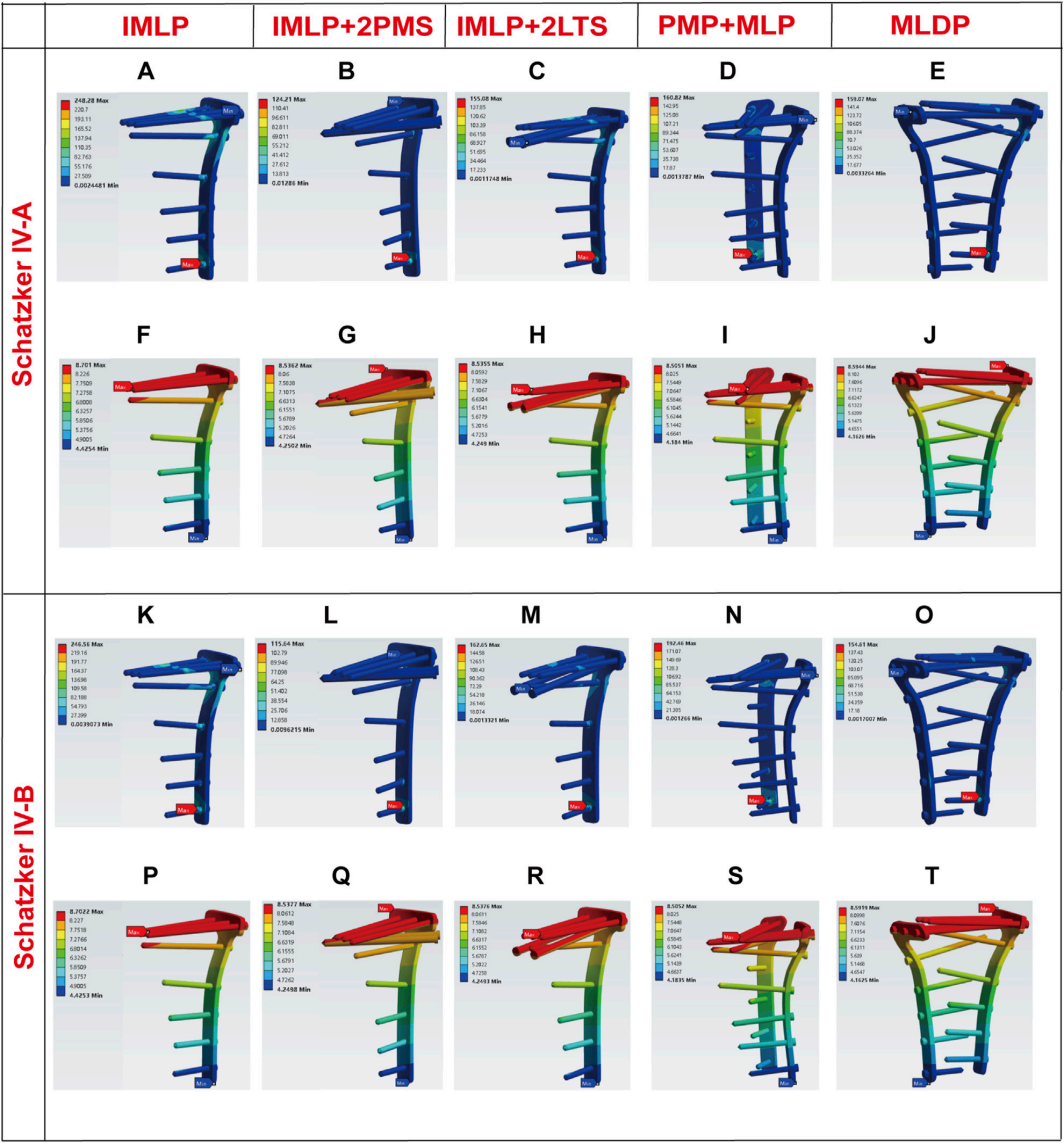
Stress and displacement nephograms of internal fixation under 1200 N axial load. **(A–E)** Stress nephograms of internal fixation for five fixation methods in Schatzker type IV-A tibial plateau fractures. **(F–J)** Displacement nephograms of internal fixation for five fixation methods in Schatzker type IV-A tibial plateau fractures. **(K–O)** Stress nephograms of internal fixation for five fixation methods in Schatzker type IV-B tibial plateau fractures. **(P–T)** Displacement nephograms of internal fixation for five fixation methods in Schatzker type IV-B tibial plateau fractures.

**FIGURE 8:**
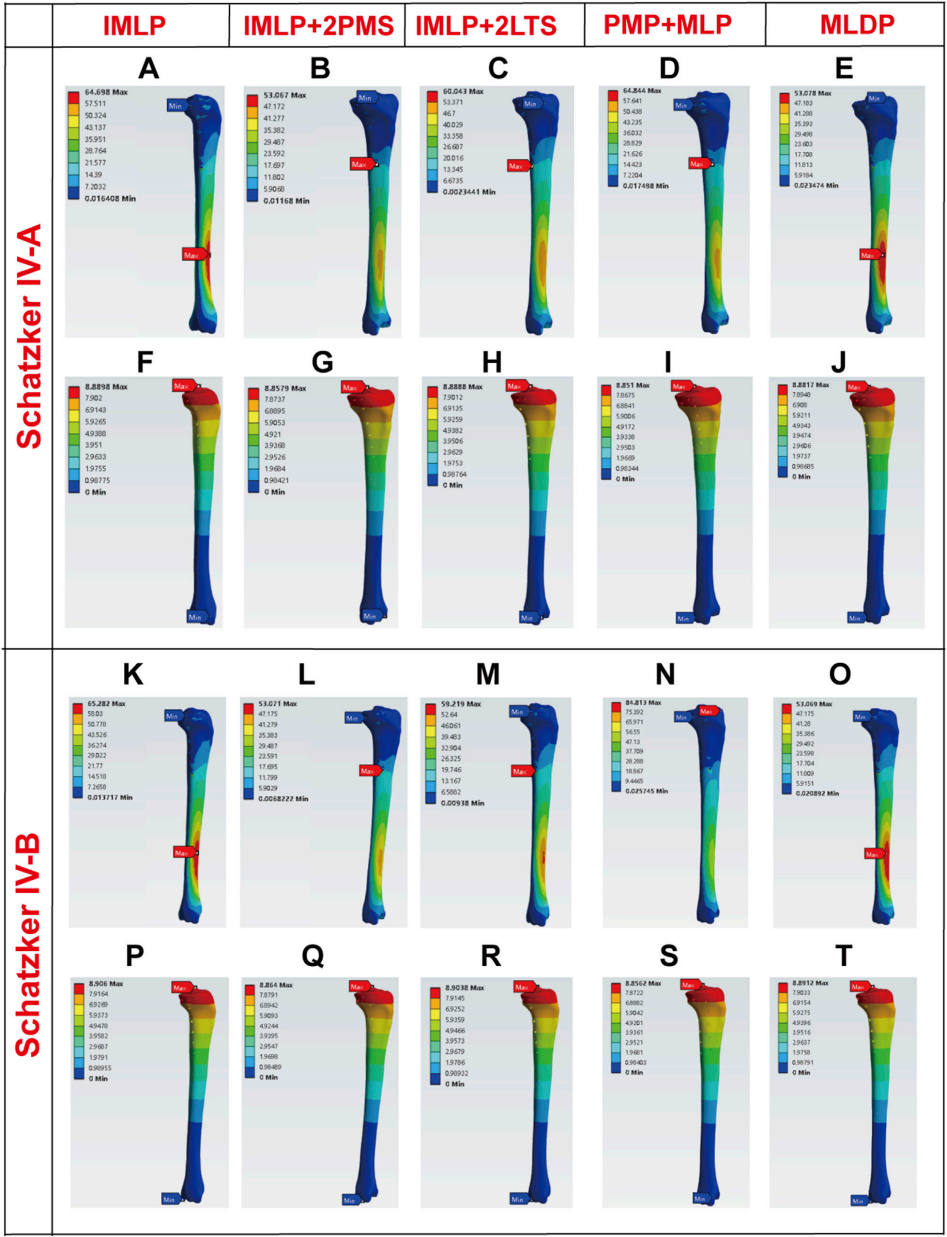
Stress and displacement nephograms of the tibial shaft under 1200 N axial load. **(A–E)** Stress nephograms of the tibial shaft for five fixation methods in Schatzker type IV-A tibial plateau fractures. **(F–J)** Displacement nephograms of the tibial shaft for five fixation methods in Schatzker type IV-A tibial plateau fractures. **(K–O)** Stress nephograms of the tibial shaft for five fixation methods in Schatzker type IV-B tibial plateau fractures. **(P–T)** Displacement nephograms of the tibial shaft for five fixation methods in Schatzker type IV-B tibial plateau fractures.

**FIGURE 9:**
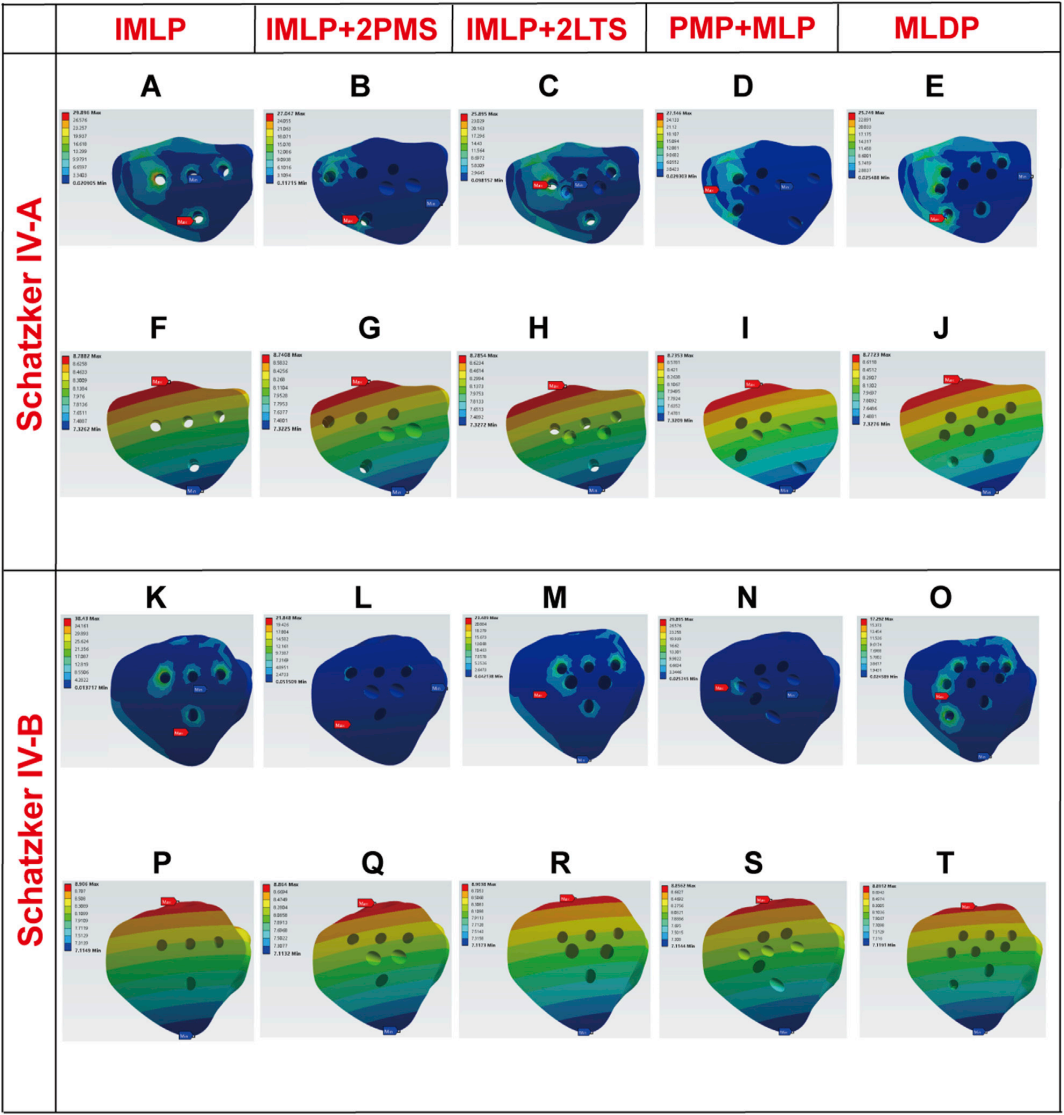
Stress and displacement nephograms of fracture fragments under 1200 N axial load. **(A–E)** Stress nephograms of fracture fragments for five fixation methods in Schatzker type IV-A tibial plateau fractures. **(F–J)** Displacement nephograms of fracture fragments for five fixation methods in Schatzker type IV-A tibial plateau fractures. **(K–O)** Stress nephograms of fracture fragments for five fixation methods in Schatzker type IV-B tibial plateau fractures. **(P–T)** Displacement nephograms of fracture fragments for five fixation methods in Schatzker type IV-B tibial plateau fractures.

**FIGURE 10:**
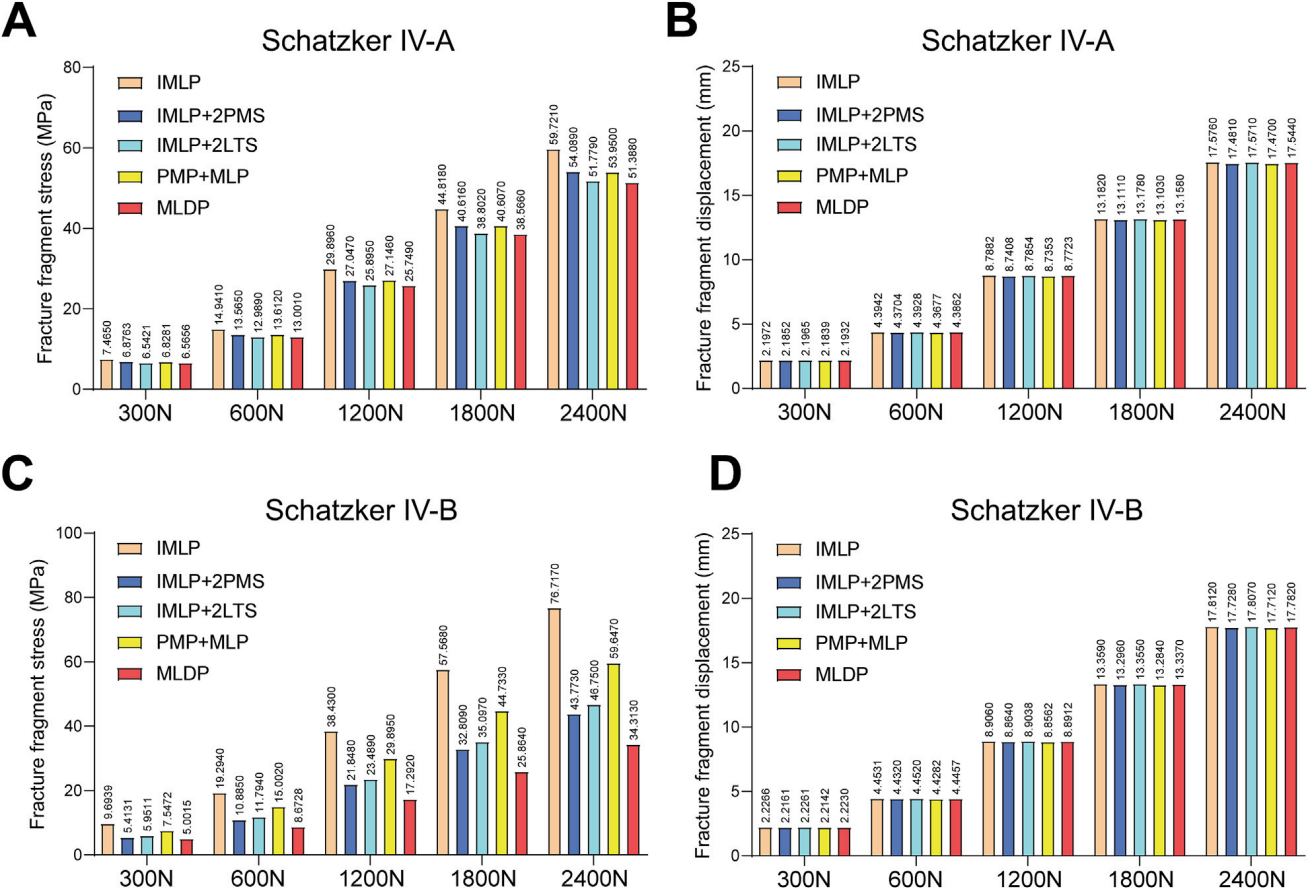
Statistical analysis of fracture fragment stress and displacement under five axial loads (300 N, 600 N, 1200 N, 1800 N, and 2400 N). **(A)** Statistical graph of fracture fragment stress for five fixation methods in Schatzker type IV-A tibial plateau fractures. **(B)** Statistical graph of fracture fragment displacement for five fixation methods in Schatzker type IV-A tibial plateau fractures. **(C)** Statistical graph of fracture fragment stress for five fixation methods in Schatzker type IV-B tibial plateau fractures. **(D)** Statistical graph of fracture fragment displacement for five fixation methods in Schatzker type IV-B tibial plateau fractures.

Figures 5, 6, 10 respectively present the peak stress statistics of the internal fixation, tibial shaft, and fracture fragments, as well as the displacement statistics, for five different internal fixation methods in the treatment of Schatzker type IV-A and IV-B tibial plateau fractures under five axial loading conditions (300 N, 600 N, 1200 N, 1800 N, and 2400 N). The numerical values for each group are indicated on the bar graphs. Figures 7, 8, and Figure 9 display the stress distribution nephograms and displacement nephograms of the internal fixation, tibial shaft, and fracture fragments under a 1200 N axial load for the five fixation methods in both Schatzker type IV-A and IV-B fractures. It can be observed that the peak stress and displacement values in all groups increased with higher loading magnitudes, while the trends of these metrics across different groups remained consistent under varying loads. The following analysis of results will focus on the data obtained under the 1200 N axial load.

### 3.1 Implant stress and displacement

Finite element analysis revealed that under a 1200 N load, the implant displacement in the five groups of Schatzker type IV-A tibial plateau fracture models, ranked from smallest to largest, was approximately: PMP + MLP (8.5051 mm) < IMLP + 2LTS (8.5355 mm) <IMLP + 2PMS (8.5362 mm) <MLDP (8.5944 mm) <IMLP (8.7010 mm). However, no significant differences were observed in the numerical values. The peak implant stress values were as follows: IMLP + 2PMS (124.210 MPa) <IMLP + 2LTS (155.080 MPa) <MLDP (159.070 MPa) <PMP + MLP (160.820 MPa) < IMLP (248.280 MPa). These data indicate that the IMLP + 2PMS group performed best in reducing implant stress, while the IMLP + 2LTS group achieved stress reduction comparable to the MLDP and PMP + MLP groups. This suggests that adding two tension screws either posteromedially or laterally to a medial plate significantly reduces the stress experienced by the implant. Figures 7A–J displays the stress distribution and displacement nephograms of the implant in the five fixation models. The peak stress consistently occurred at the connection point between the distal-most screw and the plate in the tibia.

Analysis of the internal fixation stress and displacement results for the five fixation methods under a 1200 N load in Schatzker type IV-B tibial plateau fractures (Figures 7K–T) revealed that the peak stress consistently occurred at the connection point between the distal-most screw and the plate in the tibia. The peak implant stress values were as follows: IMLP + 2PMS (115.640 MPa) <MLDP(154.610 MPa) < IMLP + 2LTS (162.650 MPa) < PMP + MLP (192.460 MPa) < IMLP (246.560 MPa). Notably, the IMLP group exhibited the highest peak stress of 246.560 MPa. In contrast, the IMLP + 2PMS group demonstrated the lowest peak implant stress (115.640 MPa), indicating its superior performance in reducing stress concentration within the implant. However, the MLDP and IMLP + 2LTS groups also achieved relatively low peak stress levels (154.610 MPa and 162.650 MPa, respectively), suggesting that supplementing a medial plate with two tension screws (either posteromedially or laterally) significantly reduces stress on the implant. Regarding fracture fragment displacement, the values were as follows: PMP + MLP (8.5052 mm) < IMLP + 2LTS (8.5376 mm) < IMLP + 2 PMS (8.5377 mm) < MLDP (8.5919 mm) < IMLP (8.7022 mm). No significant differences were observed in the numerical values, indicating that all fixation systems exhibited high overall stiffness and stability. This is critical for maintaining fracture reduction and resisting early functional loading.

### 3.2 Stress and displacement of the tibial shaft

Tibial shaft stress is a critical indicator that requires careful balance, as both excessively low and high stress levels can adversely affect fracture healing and postoperative recovery. Ideally, stress distribution in the tibial shaft should be as uniform as possible, closely resembling normal physiological conditions. For Schatzker Type IV-A Fractures under 1200 N Load: The stress and displacement nephograms of the tibial shaft for the five fixation methods are shown in Figures 8A–J. The results indicate that the tibial shaft stress in all groups aligned with the ideal state. Specifically, the peak tibial shaft stress values (from smallest to largest) were as follows: IMLP + 2PMS (53.067 MPa) < MLDP (53.078 MPa) < IMLP + 2LTS (60.043 MPa) < IMLP (64.698 MPa) < PMP + MLP (64.844 MPa). Among these, the IMLP + 2LTS group exhibited the most uniform stress distribution in the tibial shaft. Regarding tibial shaft displacement (from smallest to largest): PMP + MLP (8.8510 mm) < IMLP + 2PMS (8.8579 mm) < MLDP (8.8817 mm) < IMLP + 2LTS (8.8888 mm) < IMLP (8.8898 mm). No significant differences were observed among the groups.

For Schatzker Type IV-B Fractures under 1200 N Load: Figures 8K–O displays the stress distribution in the tibial shaft for the five fixation models. In the IMLP and MLDP groups, peak stress was located at the proximal screw holes, while in the IMLP + 2PMS, PMP + MLP, and IMLP + 2LTS groups, peak stress occurred at the inferior aspect of the tibial tuberosity. The MLDP group showed the lowest peak stress (53.069 MPa). The PMP + MLP group exhibited the highest peak stress (84.813 MPa). The IMLP + 2LTS (59.219 MPa) and IMLP + 2PMS (53.017 MPa) groups demonstrated intermediate peak stress values with relatively uniform stress distribution. No significant differences were observed in tibial shaft displacement among the groups.

### 3.3 Fracture fragment stress and displacement

Finally, we analyzed the stress and displacement of the fracture fragments (Figures 9, 10). For Schatzker Type IV-A Fractures: The peak stress values of fracture fragments (from smallest to largest) were as follows: MLDP (25.7490 MPa) < IMLP + 2LTS (25.8950 MPa) < PMP + MLP (27.1460 MPa) < IMLP + 2PMS (27.0470 MPa) < IMLP (29.8960 MPa). The MLDP and IMLP + 2LTS groups demonstrated the best performance in terms of fracture fragment stress, with values significantly lower than the compressive strength limit of cancellous bone. Notably, the IMLP + 2LTS group offered the additional advantage of being minimally invasive. Stress distribution (Figure 9C) indicated effective support of fracture fragments without significant stress concentration points. No notable differences were observed in fracture fragment displacement among the groups, suggesting that all five fixation methods provided sufficient stability for primary fracture healing.

For Schatzker Type IV-B Fractures: Detailed examination of the equivalent stress nephograms revealed that stress distribution in all models was primarily concentrated around the screw holes and the distal aspect of the fracture surface. The IMLP group exhibited the highest fracture fragment stress (38.430 MPa). The MLDP group showed the lowest fracture fragment stress (17.2920 MPa). Notably, the IMLP + 2LTS (23.4890 MPa) and IMLP + 2PMS (21.8480 MPa) groups achieved fracture fragment stress levels comparable to the MLDP group. This finding indicates that supplementing a medial plate with posteromedial or lateral tension screws effectively reduces stress concentration in the fracture fragments. No significant differences were observed in fracture fragment displacement among the groups.

## 4 Discussion

Schatzker type IV tibial plateau fractures, involving the primary weight-bearing zone (medial plateau) and carrying a high risk of soft tissue complications, remain a significant challenge in orthopedic trauma management. Unlike type IV-C fractures, which extend into the metaphysis and intercondylar eminence, type IV-A and IV-B fractures are confined to the high-load-bearing medial plateau and exhibit a significantly higher clinical incidence than type IV-C fractures (Wahlquist et al., 2007; Liu Z. et al., 2023). The primary clinical dilemma lies in balancing mechanical stability with minimally invasive techniques. This study employed finite element analysis (FEA) to systematically compare the biomechanical performance of five internal fixation strategies for type IV-A/B fractures, addressing the longstanding conflict between achieving mechanical stability and minimizing invasiveness. The results reveal critical biomechanical insights and provide quantitative evidence to support individualized fixation strategies.

### 4.1 Implant biomechanical performance: Hybrid fixation demonstrates significant advantages

The morphology and position of the tibial plateau directly influence lower limb alignment and the knee’s mechanical environment, serving as a cornerstone for maintaining joint function and stability (Tscherne and Lobenhoffer, 1993). Consequently, the mechanical stability of implants is critical for postoperative bone healing and functional recovery (Prat-Fabregat and Camacho-Carrasco, 2016; Hörmandinger et al., 2024). While double-plate fixation offers excellent mechanical stability, the extensive surgical exposure required is associated with significant trauma, increased blood loss, and higher risks of soft tissue complications (Stannard et al., 2010; Honkonen, 1994). The key finding of this study is that the hybrid fixation mode (medial plate combined with tension screws: IMLP + 2PMS/IMLP + 2LTS) achieved superior or comparable performance to traditional double-plate fixation in the core mechanical metric—implant stress—while significantly outperforming single-plate fixation. Under a 1200 N load, the IMLP + 2PMS group exhibited the lowest peak implant stress among all groups (IV-A: 124.21 MPa; IV-B: 115.64 MPa), even surpassing the traditional double-plate MLDP group (IV-A: 159.07 MPa; IV-B: 154.61 MPa). This phenomenon carries profound biomechanical implications: The medial locking plate, with its angular stability, forms the primary framework resisting axial compression forces; The posteromedial or lateral tension screws act as “neutralization screws,” effectively countering shear stresses and rotational moments in the coronal and sagittal planes. This “main load-bearing framework + auxiliary anti-slip” design effectively disperses stress concentrated at the distal end of the medial plate, significantly reducing the risk of implant failure due to metal fatigue. This provides crucial mechanical assurance for young patients expecting early weight-bearing and high-intensity functional rehabilitation.

Notably, the peak stress in all models was concentrated at the screw-plate junction in the distal tibia, indicating this region as a mechanical weak point. Clinically, optimizing screw configuration in this area (e.g., increasing screw density or utilizing locking designs) is essential to prevent mechanical failure. It must be emphasized that the inclusion of lateral double plating in this study was solely for biomechanical comparison and is not recommended as a primary clinical solution.

### 4.2 Fracture fragment stability: the critical role of posteromedial tension screws (PMS)

Stress distribution within fracture fragments serves as a direct indicator of fixation efficacy (Wang et al., 2019). Although no significant differences were observed in fracture fragment displacement across groups—demonstrating that all configurations provided initial stability—the internal stress distribution revealed the indispensable value of posteromedial tension screws (PMS). For type IV-B fractures, the IMLP + 2PMS group exhibited significantly lower fracture fragment stress (21.85 MPa) compared to the IMLP group (38.43 MPa) and approached the level of the MLDP group (17.29 MPa). This indicates that PMS plays a critical role in supporting and compressing the posteromedial fragment. The underlying mechanism lies in the fact that Schatzker type IV fractures typically result from varus-internal rotation forces, rendering the posteromedial fragment susceptible to posterior-directed tensile stresses. PMS counteracts this displacement by applying compression perpendicular to the fracture line, whereas a solitary medial plate primarily resists axial loads and offers insufficient control over shear forces. Furthermore, from the three-column theory perspective, type IV fractures often involve the posterior column, making isolated medial fixation inadequate for stability. For fractures involving both the medial and posterior columns, medial or posteromedial buttress fixation is essential to prevent reduction loss, which explains the high clinical incidence of reduction failure after isolated medial plating (Luo et al., 2010). The insertion direction of posteromedial tension screws is precisely perpendicular to the articular surface of the posteromedial fragment, enabling direct and effective support, restoring articular congruity, and resisting posterior-inferior collapse.

### 4.3 Tibial shaft stress: hybrid fixation exhibits a more physiological “stress shielding” effect

An ideal fixation system should stabilize the fracture while maintaining normal stress conduction in the tibial shaft (Aihemaiti et al., 2022). The study found that double-plate fixation (particularly the PMP + MLP group) generated significant stress concentration peaks around the proximal screw holes of the tibia (reaching up to 84.81 MPa in type IV-B fractures), which not only reflects stress shielding but also represents a potential risk factor for refracture after implant removal. In contrast, the hybrid fixation groups (e.g., IMLP + 2LTS for IV-A; IMLP + 2PMS for IV-B) demonstrated a smoother and more uniform stress distribution in the tibial shaft, closely resembling normal physiological load transfer. This characteristic of “elastic fixation” allows the bone to bear its appropriate share of the load, promoting physiological bone remodeling and healing. From a long-term perspective, this approach may yield better outcomes by reducing the risks of stress shielding-induced bone loss and refracture.

### 4.4 Classification-specific fixation strategies: differential management for type IV-A, IV-B, and IV-C fractures

**FIGURE 11:**
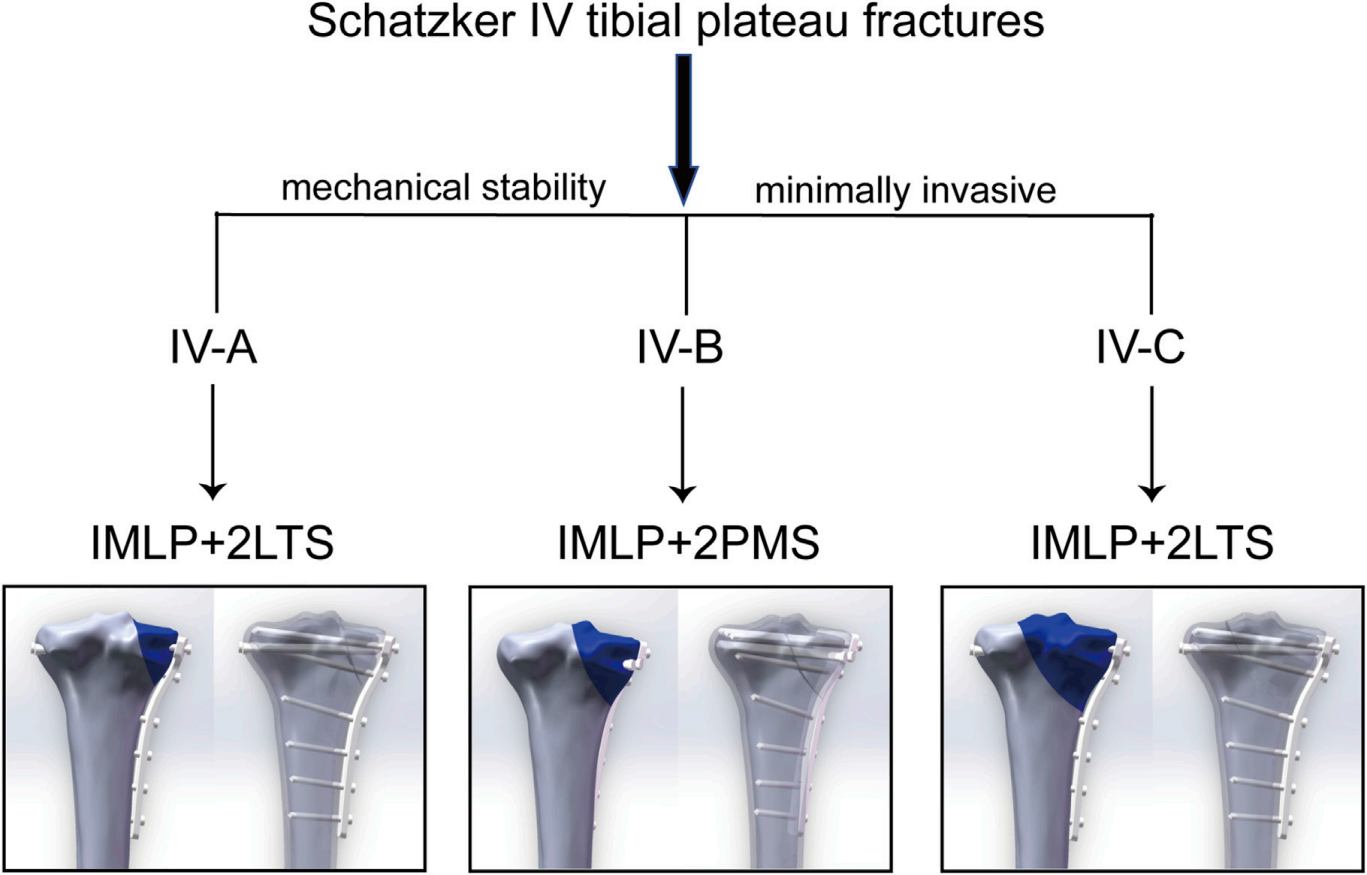
Recommended internal fixation strategies for Schatzker type IV-A/B/C fractures: Optimal solutions balancing mechanical stability and minimal invasiveness.

A key insight from this series of studies is that Schatzker type IV-A, IV-B, and IV-C fractures may require differentiated internal fixation strategies. Although both IV-A and IV-B fractures originate medial to the intercondylar eminence, their fracture line trajectories and involved column structures exhibit subtle yet critical differences. Type IV-B fractures are located more medially and directly involve the posteromedial weight-bearing area. Analysis indicates that for type IV-B fractures, IMLP + 2PMS demonstrates superior performance in reducing fracture fragment stress, while for type IV-A fractures, IMLP + 2LTS exhibits better overall stress distribution (Figure 11).

In contrast to type IV-A and IV-B fractures, type IV-C tibial plateau fractures extend laterally to the intercondylar eminence, which imposes higher demands on biomechanical stability. However, pure type IV-C fractures are relatively rare in clinical practice and are often accompanied by fractures of the lateral intercondylar eminence, posteromedial column, or partial lateral plateau. Our previous research on Schatzker type IV-C tibial plateau fractures also demonstrated that IMLP + 2LTS can enhance stability while minimizing surgical trauma (Zhou et al., 2025). This suggests that clinical decision-making should integrate the Schatzker classification, Wahlquist subtyping, three-column theory, and 3D CT imaging to meticulously evaluate fracture line patterns, the location of major fracture fragments, and posterior column involvement. A “tailored” approach to internal fixation selection—where the direction and placement of auxiliary screws are optimized based on specific fracture morphology—represents the next frontier in precision treatment.

### 4.5 Clinical translation value: Synergistic achievement of minimally invasive treatment and stability

The core clinical significance of this study lies in demonstrating that optimized fixation configurations can significantly reduce surgical invasiveness without compromising stability. First, Minimally Invasive Advantages: The IMLP + 2PMS/IMLP + 2LTS techniques require only a single incision (medial approach) combined with percutaneous screw placement, substantially reducing soft tissue dissection compared to double-plating (medial + lateral/posteromedial dual incisions). This approach significantly lowers the risk of wound complications. The reduction in soft tissue damage is not merely about incision length but fundamental differences in surgical approach: double-plating (MLDP) inevitably requires two extensive exposures—a lateral incision (e.g., anterolateral approach) and a medial incision (e.g., posteromedial approach)—both involving layered fascial dissection, muscle elevation (e.g., lifting the tibialis anterior muscle for posteromedial plating), and periosteal stripping. In contrast, hybrid fixation (IMLP with two tension screws) requires only one main medial incision for plate placement, with additional screws inserted via minimal percutaneous incisions, resulting in negligible soft tissue damage. Second, Broad Applicability: For patients with osteoporosis or high-energy trauma (poor soft tissue conditions), the hybrid fixation method (medial plate with screws) avoids extensive exposure, reduces the risk of periosteal blood supply disruption, and FEA-confirmed mechanical reliability supports early weight-bearing. This makes it an optimal choice for elderly patients and those with soft tissue injuries. Finally, efficiency and cost-effectiveness: Reducing the number of plates used can shorten surgical time and lower material costs.

### 4.6 Limitations and future directions

This study has several limitations. First, the finite element analysis (FEA) model was based on linear elastic material assumptions, which cannot simulate biological changes during bone healing or material plastic deformation. Second, the impact of bone quality variations was not systematically evaluated. Future work will employ parametric modeling methods (e.g., adjusting cortical bone thickness by ±20% and trabecular bone elastic modulus by ±30%) to quantify the range of mechanical performance fluctuations. Additionally, the Schatzker type IV-A/B fractures investigated in this study correspond to the “extension-varus” pattern within the three-column classification system. Further comprehensive analysis is needed for flexion-varus and hyperextension-varus fracture types. Moreover, segmentation, fracture modeling, and implant placement were strictly performed by four co-authors under the guidance of the two corresponding authors according to the experimental protocol. While this ensured clinical relevance, formal inter-observer variability assessment has not been conducted and represents a potential direction for future research. Finally, the finite element model was constructed based on CT data from a single typical patient. Although it effectively reflects the core biomechanical mechanisms of Schatzker type IV fractures, it must be acknowledged that individual differences (such as bone density and fracture comminution) may affect the generalizability of the results. Future studies should incorporate diverse geometric models (including subgroups with osteoporosis and high-energy comminuted fractures) to further validate the robustness of fixation strategies.

Future research will focus on: 1) Developing more sophisticated nonlinear finite element models that integrate muscle forces and dynamic loading conditions; 2) Conducting cadaveric biomechanical experiments to validate the FEA conclusions; and 4) Implementing multicenter, prospective randomized controlled trials (RCTs) to empirically test the superiority of this classification-guided optimized fixation strategy across biomechanical, radiological, and patient-reported functional outcomes.

## 5 Conclusion

In summary, this study, through comprehensive finite element analysis, confirms that the hybrid fixation strategy of “medial plate combined with tension screws” represents a biomechanically optimal solution for the treatment of Schatzker type IV tibial plateau fractures. It not only excels in key metrics such as implant stress, fracture fragment stability, and tibial shaft stress distribution, but also successfully reconciles the seemingly contradictory goals of minimally invasive intervention and rigid fixation. This research underscores the importance of subtype-specific analysis for type IV fractures and the subsequent formulation of personalized fixation strategies. By leveraging finite element analysis, we demonstrate that hybrid fixation achieves a balance between stability and safety through minimal invasiveness, offering an ideal solution for patients at high risk of soft tissue complications (e.g., elderly or high-energy trauma cases). For type IV-A fractures, characterized predominantly by medial plateau split, we recommend IMLP + 2LTS (lateral tension screws) to optimize anti-rotational stiffness. For type IV-B fractures, which involve the intercondylar eminence, IMLP + 2PMS (posteromedial tension screws) is the preferred choice to enhance axial support. The findings of this study provide evidence-based support for the precise management of Schatzker type IV fractures, facilitating a shift in clinical practice from “experience-driven” to “biomechanically optimized” decision-making. Ultimately, this approach aims to improve functional outcomes for patients.

## Data Availability

The raw data supporting the conclusions of this article will be made available by the authors, without undue reservation.

## Supplementary material

SUPPLEMENTARY FIGURE S1 Comparison of soft tissue injury associated with surgical incision sizes of different internal fixation methods. **(A)** Surgical incision size and soft tissue damage range for medial-lateral double plating (MLDP) in the treatment of tibial plateau fractures. **(B)** Surgical incision size and soft tissue damage range for posteromedial plating combined with lateral tension screws in the treatment of tibial plateau fractures.

SUPPLEMENTARY FIGURE S2 Minimally invasive advantages and cost-effectiveness of hybrid “plate + screw” fixation. **(A)** Postoperative X-ray of tibial plateau fracture treated with medial-lateral double plating. **(B)** Postoperative X-ray of tibial plateau fracture treated with posteromedial plate combined with lateral tension screws. The results demonstrate that the “plate + screw” hybrid approach not only achieves minimally invasive effects but also reduces costs and shortens surgical time.
