# Supplementary figures and images for: Breaking the treatment dilemma of Schatzker IV fractures: finite element analysis validates hybrid single-plate with tension screw fixation for synergistic optimization of stability and minimally invasive outcomes
Source: Front Bioeng Biotechnol. 2025 Oct 31;13:1650132. doi: 10.3389/fbioe.2025.1650132 (PMC12615468; doi:10.3389/fbioe.2025.1650132)

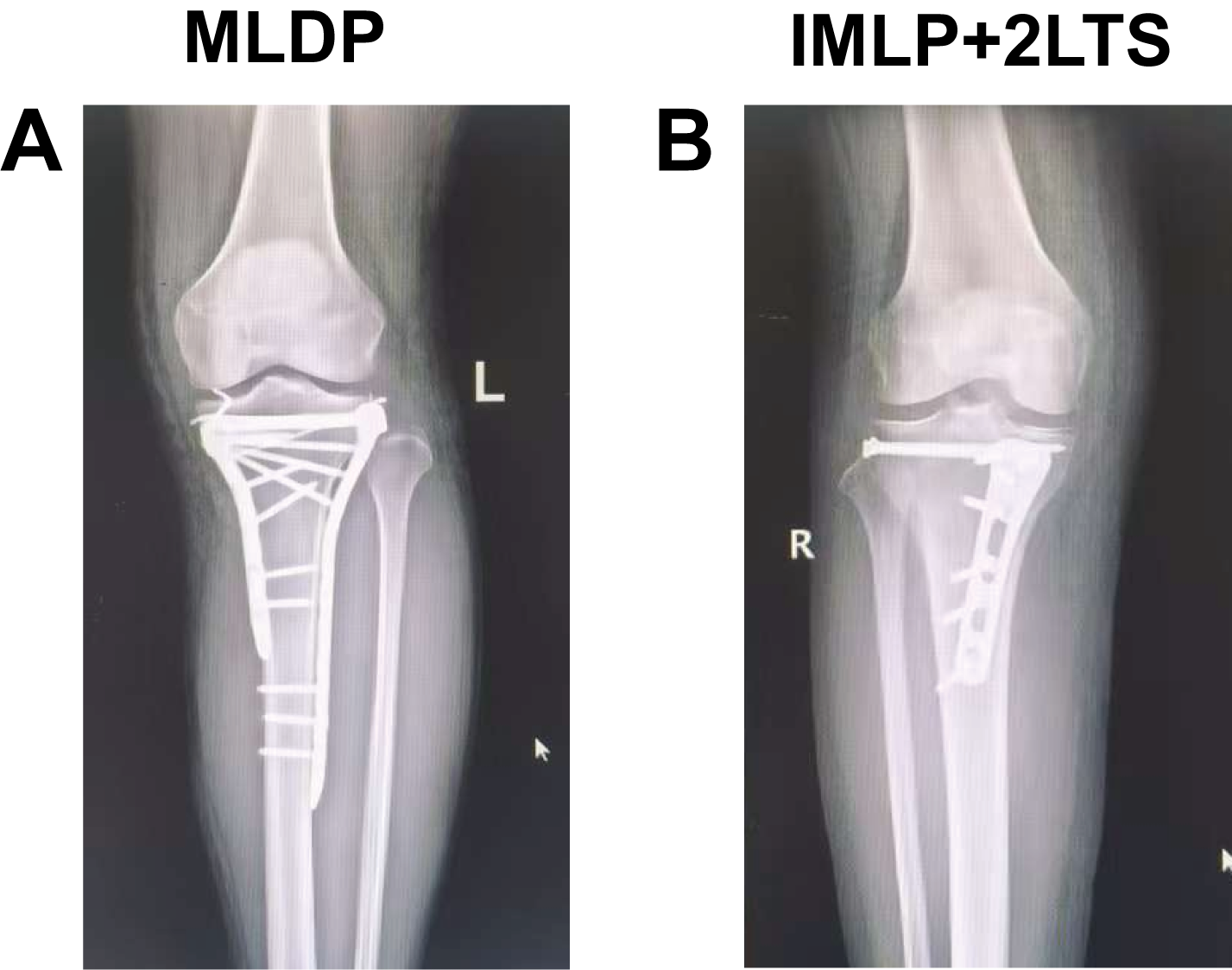

Supplement: Supplementary file 1 [file Image2.tif]

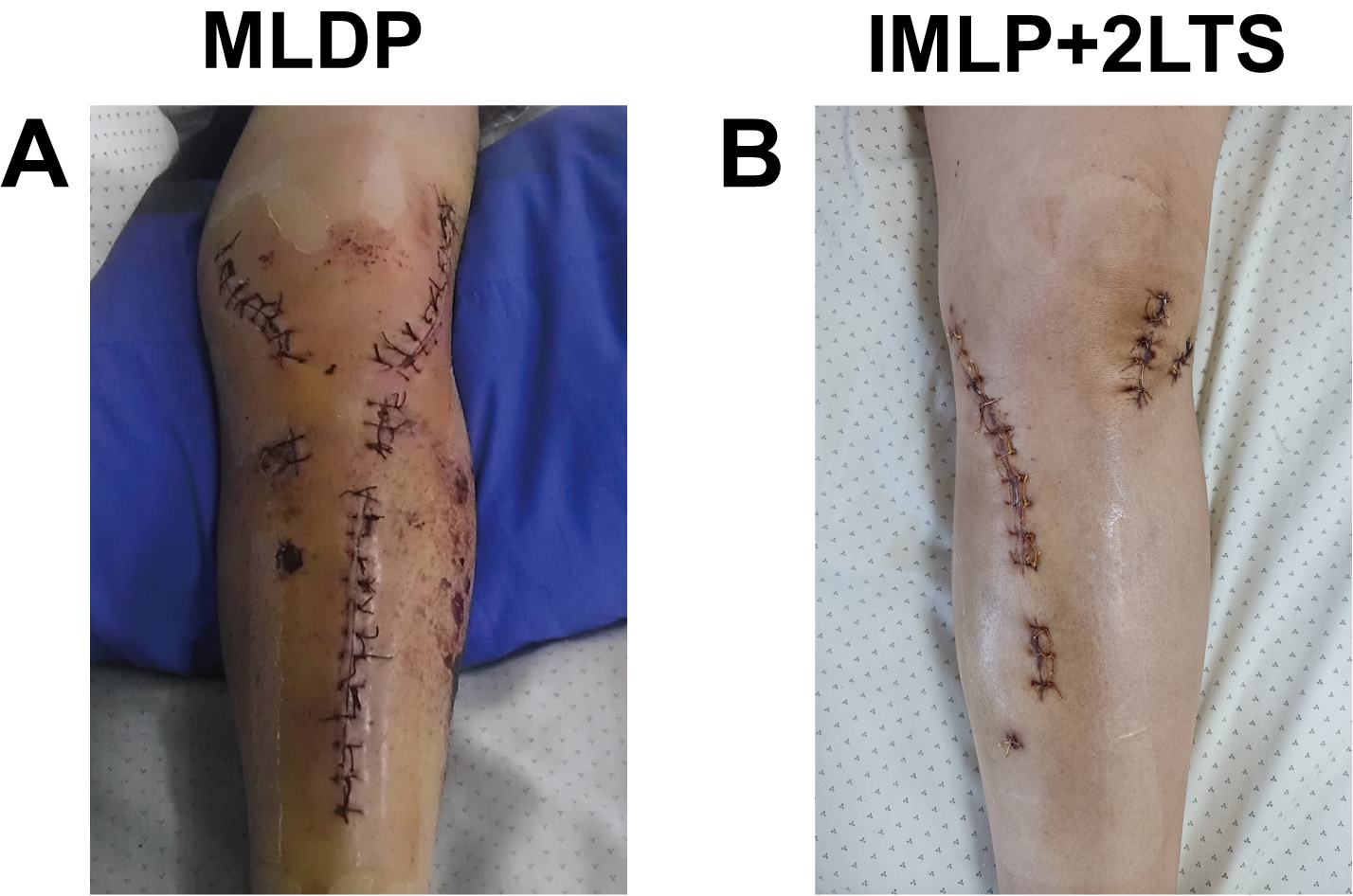

Supplement: Supplementary file 2 [file Image1.tif]
